# The Reactive Oxygen Species in Macrophage Polarization: Reflecting Its Dual Role in Progression and Treatment of Human Diseases

**DOI:** 10.1155/2016/2795090

**Published:** 2016-04-06

**Authors:** Hor-Yue Tan, Ning Wang, Sha Li, Ming Hong, Xuanbin Wang, Yibin Feng

**Affiliations:** ^1^School of Chinese Medicine, Li Ka Shing Faculty of Medicine, The University of Hong Kong, Pokfulam, Hong Kong; ^2^Laboratory of Chinese Herbal Pharmacology, Renmin Hospital, Hubei 442000, China; ^3^Hubei Key Laboratory of Wudang Local Chinese Medicine Research, Hubei University of Medicine, Hubei 442000, China

## Abstract

High heterogeneity of macrophage is associated with its functions in polarization to different functional phenotypes depending on environmental cues. Macrophages remain in balanced state in healthy subject and thus macrophage polarization may be crucial in determining the tissue fate. The two distinct populations, classically M1 and alternatively M2 activated, representing the opposing ends of the full activation spectrum, have been extensively studied for their associations with several disease progressions. Accumulating evidences have postulated that the redox signalling has implication in macrophage polarization and the key roles of M1 and M2 macrophages in tissue environment have provided the clue for the reasons of ROS abundance in certain phenotype. M1 macrophages majorly clearing the pathogens and ROS may be crucial for the regulation of M1 phenotype, whereas M2 macrophages resolve inflammation which favours oxidative metabolism. Therefore how ROS play its role in maintaining the homeostatic functions of macrophage and in particular macrophage polarization will be reviewed here. We also review the biology of macrophage polarization and the disturbance of M1/M2 balance in human diseases. The potential therapeutic opportunities targeting ROS will also be discussed, hoping to provide insights for development of target-specific delivery system or immunomodulatory antioxidant for the treatment of ROS-related diseases.

## 1. Introduction

Accumulating studies have implied the physiological role of reactive oxygen species (ROS) in various biological processes at distinct levels, for example, gene expression, protein translation, posttranslational modification, and protein interactions. Instead of merely being a harmful byproduct of metabolism, cell-derived ROS majorly derived from hydrogen peroxide (H_2_O_2_), superoxide anions (O_2_^−^), and hydroxyl radicals (OH^−^), is an independent or cooperative regulator for cellular signalling in response to environmental cues. For instance, H_2_O_2_, due to its long-lived nature and ability to easily pass through cellular membrane, is an important secondary messenger in maintaining the cellular homeostasis under different conditions [1]. It may result in activation or shutdown of diverse cellular processes such as cell cycling, chromatin remodeling, DNA repair, cell differentiation, and self-renewal [2]. The balance of intracellular ROS is therefore extremely important in maintaining normal physiology of human beings. While mitochondrial respiratory chain is the major component that cells used to produce intracellular ROS, cells develop a series of antioxidant enzymes to reduce redundant ROS. These enzymes, including catalase, superoxide dismutase (SOD), glutathione peroxidase (GPx), and glutathione reductase (GR), can either grossly or specifically catalyze different types of ROS. The presence of other endogenous antioxidants such as peroxiredoxins and thioredoxins ensures critical monitoring of cellular ROS level and conserves the normal redox homeostasis. In some pathological conditions, the redox balance is disturbed when the intracellular redox system is shifted to oxidized state, which is defined as oxidative stress. During oxidative stress, cells produce oxidized lipids, proteins, and damaged DNA which would consequently lead to cell death and tissue damage. In this regard, sustained oxidative stress has been linked with a series of human progressive diseases such as hepatitis, diabetes, and cancers.

Macrophages are long-lived innate immune cells that ubiquitously populate in almost all tissues. The general source of macrophages is bone marrow derived monocytes, which migrate to various tissues and adhere to become mature macrophages. However, studies also revealed that macrophages can be presented bypassing monocytic stages. In many human diseases, macrophages detect tissue injury or infections and process the damage or remodeling of wounded tissues [3]. Macrophages also sense the unfavourable conditions within human body including hypoxic and metabolic stresses where response of host defense and immune regulation are triggered [4, 5]. All these propose macrophages as an important regulator in multiple biological processes, including innate and adaptive immunity, angiogenesis, reproduction, and even malignancy [6]. The multiple function of macrophage is facilitated by its high plasticity in response to environmental- or self-derived stimulating signals [7]. This macrophage heterogeneity is reflected by functional polarization of differentiated macrophages and the dynamic switch between phenotypes. Under physiological condition, the M1/M2 population remains in mixture state while disoriented shift from M1 to M2 or vice versa results in disease progression. Multiple signalling pathways and several cellular stimuli, such as cAMP [8], phospholipid [9], and irons [10], have been found involved in the functional polarization of macrophages. To our knowledge, there are not many studies that highlighted the essential role of ROS in regulating the functional polarization of macrophages. Thus, in this review, we discussed the involvement of ROS in macrophage reprogramming. The biology of macrophage polarization, disturbance of M1/M2 balance in human diseases, and the role of ROS in macrophage homeostatic functions were addressed. How ROS drives macrophages polarization and the potential therapeutic opportunities targeting ROS were specifically reviewed and discussed.

## 2. Biology of Macrophage Polarization

One of the important hallmarks of macrophage is its high heterogeneity, allowing them to be activated to different functional statuses with particular properties upon exposure to endogenous and exogenous inducers in the microenvironment. Mirroring T_H_1/T_H_2 programming, macrophage could be reprogrammed to classically activated (M1) and alternatively activated (M2) subsets in response to the surrounding stimuli. Classically activated M1 macrophages are characterized by its high microbicidal function associated with the ability to secrete high amount of proinflammatory cytokines such as interleukin-1*β* (IL-1*β*), IL-12, tumor necrosis factor-*α* (TNF-*α*), and superoxide anions. As to alternatively activated M2 macrophages, their functionality is distinct from the classical one which produced high levels of anti-inflammatory mediators IL-10 and tumor growth factor-*β* (TGF-*β*) and express cell surface markers such as mannose receptor (MR/CD206) and scavenger receptor (SR/CD163), which mainly participate in tissue remodeling, parasitic clearance, and resolution of inflammation. In healthy subject, macrophage remains in M1/M2 mixture state; therefore macrophage polarization is important to determine tissue fate. The switch of macrophages phenotypes towards extreme state of either M1 or M2 over time may result in several disease progressions such as obesity, cancer, and rheumatoid arthritis. Therefore, understanding the functionality of M1 and M2 macrophages and its role in disease progression may be crucial.

### 2.1. M1: The Classically Activated Macrophage

The major homeostatic function of naive macrophages is clearance of apoptotic debris, produced during the cellular process. In response to various endogenous danger signals, macrophage physiology is altered followed by production of proinflammatory mediators and modification of surface markers. Classical M1 macrophages could be activated by either interferon-*γ* (IFN*γ*) secreted by NK cells and adaptive T helper 1 (T_H_1) cells or pathogen-associated molecular patterns (PAMPs) released by microorganisms through Toll-like receptor (TLRs) via MyD88 dependent manner [11]. Meanwhile, stimulation of TLR3 and TLR4 may also activate MyD88 independent pathway that induces IFN*β* secretion. This pathway is mediated by Toll/IL-1 receptor (TIR) domain-containing adaptor and knockdown of TIR led to blockade of TLR4 mediated inflammation [12].

The activation of classical M1 macrophages induces large amount of inflammatory genes and chemokines secretion which facilitates the antigen presentation and recruitment of T_H_1 response for subsequent pathogen killing activity. Recent studies established the metabolic characteristics for M1 and M2 macrophages, suggesting that the macrophage metabolism under pathological condition also governs the functional phenotypic changes of macrophages [13]. Activation of M1 macrophages is associated with upregulation of iron storage protein H ferritin and reduction of ferroportin, leading to iron retention and inflammation [14]. The glycolytic flux is favourable in classical M1 macrophages phenotype that involves the expression changes of 6-phospho-2-kinase isoforms from liver-type to more active ubiquitous isoenzyme, thereby maintaining the level of fructose-2,6-bisphosphate [15]. Besides, M1 macrophage is associated with higher aerobic glycolysis and extracellular acidification rate [16]; increase of HIF-1*α* further associates with IL1*β* promoter and maintains IL1*β* production in M1 macrophages. Through regulating glycolytic flux, blockade of carbohydrate kinase-like protein CARKL triggers M1 polarization [17] while pyruvate dehydrogenase kinase 1 PDK1 promotes aerobic glycolysis in M1 macrophages [18]. Apart from glucose metabolism, activation of M1 macrophages is accompanied by increase of COX-2 and reduction of COX-1, thromboxane A synthase 1, arachidonate 5-lipoxygenase, and leukotriene A4 hydrolase [19].

### 2.2. M2: The Alternative Activated Macrophage

In contrast to classical activated macrophages, innate immune cells such as basophils and mast cells and other adaptive cells produce IL-4 and IL-13 priming M2 alternative phenotype. IL-4 induced M2 macrophages expressed high concentration of IL-10, decoy receptor IL-1R, IL-1R antagonist, chemokines CCL22 and CCL17, and intracellular enzyme arginase-1. All of these ensue the recruitment and activation of T_H_2 immune response and immune-suppressive function of M2 macrophages. In addition to T_H_2 immune response, IL-4 induced macrophages stimulate arginase activity by converting arginine to polyamines and collagen precursors that are crucial for tissue modeling and wound healing. M2 macrophages also produce VEGFA, EGF, and IL-8 that are responsible for angiogenesis and lymphangiogenesis [7]. Apart from IL-4 induced phenotype, different schemes of M2 macrophages classification have been proposed due to the overlapping properties of alternative activated macrophages. The activation of M2 macrophages stimulated by addition of either IL-4 and IL-13, TGF*β*, immune complexes, glucocorticoids, or IL-10 may yield distinct activation profiles [20].

Cellular metabolism especially lipid metabolism also incurs important role in providing energy fuel for activation of alternative M2 macrophages. In iron metabolism, M2 macrophages increased ferroportin expressions, which further induced iron export [14]. As opposed to M1 classical activation, M2-regulated gene transcription occurs in condition favouring of mitochondrial metabolism and oxidative glucose metabolism, in which the M2 phenotype tends to be switched towards proinflammatory state under low oxygen condition [16]. In lipid metabolism, M2 macrophage activation is associated with fatty acid oxidation and its uptake. M2-secreted lysosomal acid lipase as well as its scavenger receptor CD36 facilitates uptake of triglycerols, LDL, and VLDL for *β*-oxidation and fatty acid generation [21]. Apart from LDL and VLDL, high density lipoproteins promote anti-inflammatory function through ATF3 dependent pathway [22]. Study by Prieur et al. [23] also postulated that short and saturated fatty acids favour M1 polarization, while longer and unsaturated fatty acids induce M2 anti-inflammatory phenotype. Therefore, M2 activation is accompanied by upregulation of arachidonic acid [19], omega-3 polyunsaturated fatty acids [24], sphingosine 1-phosphate [25], eicosapentaenoic acid, and docosahexaenoic acid [26]. All the correlation of alternative M2 activation with lipid metabolism also provides clue for the important role of M2 activation in atherosclerosis, obesity, and insulin sensitivity (as discussed later).

### 2.3. Disruption of M1/M2 Balance in Human Diseases

Classical M1 macrophages elicit major role in inducing inflammation and clearing of pathogens, whereas alternative M2 macrophages resolve inflammation and are crucial for the functions of tissue modeling and wound healing. They represent the opposing ends of the activation spectrum, and either accumulation of M1/M2 signals may lead to deterioration of the host. Extensive production of IL1, IL6, and IL23 by M1 macrophages incurs activation of T_H_17 immune response and leads to autoimmune disorder progression [27]. Earlier studies may postulate that macrophages populations promote disease progression but many recent studies have proposed that the proportions of different macrophage phenotypes contribute to disease progression. There is no absolute answer on which phenotype is “good” or “bad” as the switch of resident or recruited macrophages towards M2 phenotype may trigger tumor progression while accumulation of M1 macrophages leads to insulin resistance and atherosclerosis. Therefore, continuous activation of either states brings harm to the host and the understanding of how disruption of M1/M2 in regulating diseases may be essential. The involvement of M1/M2 in obesity, atherosclerosis, and cancer is briefly discussed below; the comprehensive reviews on human diseases have been published elsewhere [28–30].

#### 2.3.1. Obesity and Insulin Resistance

Adipose tissue macrophages (ATM) are predominantly alternative M2 phenotype in lean subjects, and the ATM are reprogrammed to classically activated M1 phenotype with proinflammatory function upon exposure to free saturated fatty acids produced by adipose tissue, resulting in reduced insulin sensitivity in white adipose tissue. IL-10 secreted by M2 macrophages may be the responsible cytokine that protects adipocytes from obesity leading insulin resistance [31]. The free fatty acids will also trigger production of CCL2 by adipocytes and ATM in order to promote the recruitment of proinflammatory monocytes to the inflamed tissue [32]. Knockout of CCR2 in murine model effectively ameliorated the adipocyte inflammation, macrophage infiltration, and insulin resistance in obese mice [33].

#### 2.3.2. Atherosclerosis

Atherosclerosis develops with the accumulation and trapping of low density lipoproteins (LDL) in the intima of the arteries, and the modified LDL-induced secretion of adhesion molecules by endothelial cells recruits monocytes with proinflammatory phenotype to atherosclerotic lesion, which eventually differentiate into lipid-laden foam cells [34]. Cholesterol crystal, IFN*γ*, LPS, and oxidized LDL are known to stimulate M1 activation and M1-activated cytokines further support the monocyte recruitment and macrophage retention at the plaque area [35]. Due to the involvement in plaque destabilization, the proatherogenic M1 populations are predominantly accumulated at the plaques where they are prone to rupture. As for M2 macrophages, they mostly reside at stable-cell enriched plaque and adventitia. M2 macrophages tend to prevent foam cell formation and protect against atherosclerosis.

#### 2.3.3. Cancer

The growth and expansion of malignant cells is complex, which involves gene manipulation as well as establishment of microenvironment that favours tumor progression. Many recent studies have highlighted the role of tumor-associated macrophages in promoting cancer growth, angiogenesis, invasion, migration, and T cell suppression [36]. In most cancers, macrophages residing in tumor microenvironment exhibit M2 phenotype with immunosuppressive property. In contact with tumor cells, M2 macrophages tend to derive certain substances such as various growth factors, chemokines, and proteases that maintain tumor growth and expansion [37]. Apart from mediating tumor growth and progression, M2 macrophages interact with other immune cells and suppress innate and adaptive antitumor immune response. Depending on the tumor environment, phenotype of tumor-associated macrophages would be reprogrammed to M1-like phenotype, which is characterized by the antigen presenting property and tumoricidal function that favours tumor regression [38]. M1 macrophages activation increases expressions of mediators that are responsible for antigen presentation and costimulation; this may further promote infiltration of neutrophils to the tumor area leading to neutrophil-targeted tumor regression [39].

## 3. Reactive Oxygen Species on Homeostasis Function of Macrophages

### 3.1. ROS in Regulating Phagocytosis of Macrophages

Numerous studies have unveiled the diverse regulations of ROS on the phagocytosis function of macrophage. The regulation may be controversial, but the discrete role of ROS on macrophages may be impacted by the different sources of ROS as well as plasticity of macrophage itself, which would be discussed in detail below. As macrophages are endogenous scavengers for dying cells in various pathological conditions, interaction between macrophages with compartments determines the phagocytic function of macrophages. Dying cells produce high levels of ROS, which are released into extracellular area when cellular membrane is degraded during cell death. Attachment of dying cells to macrophages requires intercellular communication in which ROS may play a role. On the other hand, extracellular and intracellular ROS may differentially control the phagocytosis process of macrophage by regulating the ability and capacity of macrophages in the uptake and degradation of dying compartments. In this regard, ROS plays a critical regulatory role in determining the initiation and outcome of cellular phagocytosis.

#### 3.1.1. The Role of ROS from Dying Cells during Phagocytosis

Engulfment of cells undergoing apoptotic programmed cell death (PCD) by macrophage is initiated by the presentation of membrane signals to phagocytes that allows recognition of dying compartments. In macrophage-driven phagocytosis, these molecules include scavenger receptors, for example, CD36, immunoglobulin super-family molecules, CD31, complement receptors, such as C91, sugar and phospholipid-engaging molecules, for example, lectins and PSR, and some integrins such as *α*_v_ *β*_3_. The corresponding components on cellular membrane of apoptotic cells were less identified, but structures encompass lipid, carbohydrate, and protein which are exposed as molecules presenting find-me signals towards extracellular area during cell apoptosis [40]. These lipids and proteins presented on membranes of apoptotic cells are generally regarded as substrates of oxidation reaction containing dying cell-produced ROS. Indeed, experimental evidence has shown that oxidation of lipids and proteins by ROS confers recognition and attachment of macrophage towards apoptotic cells. By using monoclonal antibody that blocks the epitopes of oxLDL of apoptotic cells, scientists observed failure of engulfment of dying cells by murine peritoneal macrophages [41]. It was further observed that surface presentation of oxidative modified phosphatidylserines (PS) on apoptotic cells is essential for macrophage engulfment [42]. The phagocytosis was blocked upon presence of lipoprotein-associated phospholipase A2 (Lp-PLA2), a secreted enzyme with high specificity towards PS metabolites. Oxidation of PS is often observed during cell apoptosis [43]. This gives rise to the mechanism underlying attenuated phagocytosis of etoposide-treated cells by macrophage as etoposide is able to suppress oxidation and externalization of PS of the apoptotic cells [44]. In addition, some phosphatidylcholine (PC) species, which are oxidized during early- to late-apoptotic process, present to the surface of apoptotic cells are recognized by C-reactive proteins, facilitating clearance of apoptotic cells by macrophage engulfment [41]. Study revealed that oxidation of PC (oxPC) was majorly processed by ROS, in virus-infected cells, and scavenge of ROS by NAC abrogated oxPC production [45]. Although macrophage receptors correspond to particular type of oxidized lipids and proteins have not been fully unveiled, all these studies have paved the importance of ROS-driven oxidation reaction in dying cells during initiation of phagocytosis.

#### 3.1.2. The Role of Macrophage ROS in Regulating Phagocytosis

Besides dying cells, macrophage itself also produces intracellular ROS that is involved in the phagocytic process. It is a notion that ROS in macrophage is essential for uptake and clearance of apoptotic cells; however, maintaining high level of ROS may be harmful to macrophage as, in some studies, inducible ROS is sufficient to cause macrophage apoptosis [46]. Hypothesis of adaptive mechanism underlying survival of macrophage in high ROS condition was ever discussed, which included increase expressions of DNA repair proteins [47] and endogenous antioxidative enzymes [48] during monocyte-macrophage differentiation and classical activation of macrophage. Production of ROS by macrophage majorly relies on Nox2 gene, and study showed that activation of Nox2 gene in murine macrophage cell line RAW264.7 as well as primary peritoneal macrophages by carotenoid lutein induced ROS production that was responsible for the increased phagocytic activity [49]. Additionally, production of mitochondrial ROS (mROS) is able to increase phagocytosis of macrophages. In mROS-driven phagocytosis, intracellular fatty acids are utilized as fuel for oxidative phosphorylation by mitochondria-localizing enzyme encoded by Immunoresponsive gene 1 (IRG1). *β*-oxidation of fatty acids is associated with mROS production and augments the bactericidal activity of macrophages [50]. These observations give rise to the critical role of intracellular ROS in clearance of apoptotic cells by phagocyte. Besides, NO produced by phagocytosing macrophages is important for PS externalization of dying cells [51]. And this was further proved by the notion that phagocytosis entry requires class I PI3K product phosphatidylinositol 3,4,5-trisphosphate-induced ROS production in murine macrophages [52]. In fact, phagocytosis of macrophages requires Nox2-dependent production of extracellular ROS [53], and clinical evidence of essential role of ROS was noted in chronic granulomatous disease patients, who lack Nox2, owning macrophages that failed to efficiently engulf apoptotic cells [54].

Additionally, oxidative burst is also required in the clearance of apoptotic cells by alternatively activated macrophages during wound healing process [53]. However, the increase of ROS in macrophage during early stage of apoptotic cell clearance is followed by attenuation of oxidative burst by PPAR*γ* activation. In PPAR*γ*-mutant macrophages, ROS level was restored due to constituting activation of PKC pathway during apoptotic cell clearance [55]. Further study revealed that resolvin D1 (RvD1), one of endogenous proresolving lipid mediators derived from docosahexaenoic acid, is able to inactivate Nox2 and inhibits production of ROS after engulfment of apoptotic cells by macrophages, which prevents macrophage from apoptotic cell death [56]. This indicates that ROS increase in macrophage is transient during phagocytic process of apoptotic cells. Indeed, intracellular level of ROS within macrophages can be triggered by cells undergoing apoptosis or necrosis. This oxidative burst system helps macrophages to recognize necrotic cells whose clearance requests an inflammatory reaction. In necrosis-related cell death, dying cells release high concentration of high-mobility group box1 (HMGB1), which triggers inflammatory response in macrophages [57]. Other molecules like calgranulins and adenosine triphosphate derived from necrotic cells trigger Nox2 activation in macrophage and produce more ROS [58, 59]. On the contrary, engulfment of apoptotic cells inhibits persistent ROS production thereby preventing activation of a secondary inflammation that is harmful to any bystander cells. This indicates a host response of engulfed compartment in the extent of oxidative burst. On the other hand, pathogens are able to develop mechanism in responding to macrophage-derived ROS, which was observed from the induced arginine-biosynthetic genes in* C. albicans* [60]. In summary, the phagocytosis process of macrophage can be regulated by ROS, which involves responses of host and engulfed cells towards oxidative burst.

### 3.2. ROS in the Control of Death of Macrophage

Increase of ROS during the differentiation and phagocytosis of macrophages may be harmful to the cells. Although increased levels of DNA repair proteins and ROS reductase in macrophage make it become highly ROS-resistant in fighting against overwhelmed intracellular ROS level [47, 48], it is still not completely prevented from ROS-associated death. Death of macrophage may not be favourable during diseases treatment, as dying macrophages may induce secondary response of necrotic cell death, which includes release of proinflammatory cytokines and proteolytic factors that further activate inflammation [61]. High level of circulating heme has been demonstrated to induce necrotic cell death of macrophage, which is associated with increased intracellular ROS level. And such macrophage death further affects the intracellular infection control of, for example, malaria and sepsis [62]. Study has revealed that a tissue damaging agent methemoglobin has toxicity towards murine macrophages by increasing the ROS production. In this case, peripheral presentation of methemoglobin may lead to multiple tissues damage as well as immunosuppression [63]. These observations reveal that an overwhelmed ROS level in macrophage may lead to diseases-associated cell death. In fact, induction of cell death by ROS can be primarily due to Nox2 activity on phagosomes [64], as well as secondary responses towards several extracellular and intracellular factors. It was found that extracellular oxidatively modified high density lipoprotein induces ROS level in human-derived macrophage lineage cells, which is associated with macrophage death [65]. This was similarly observed in human-derived macrophages treated with oxidatively modified low density lipoproteins (oxLDL) [66], the mechanism of which may involve activation of Nox by lysophosphatidylcholine, a side product of LDL oxidation [67]. Interestingly, the cell death-inducing effect is not observed in either naive HDL or LDL. The phenomenon is commonly observed in atherogenesis, in which the persistent macrophage foam cell death and efferocytosis drive the formation of advanced lesions. Other factors that could result in ROS-associated cell death in macrophage include cytokines [68] and free cholesterol [69]. Additionally, some studies revealed that ROS-associated ER stress may lead to macrophage death upon being challenged by* Mycobacterium tuberculosis* and* Mycobacterium kansasii* [70, 71], which may be related to the presence of their heparin-binding haemagglutinin antigen [72], indicating that the ER stress may be the downstream event of elevated ROS in dying macrophage. Increased ER stress subsequently altered calcium homeostasis and activated Nox-mediated ROS formation, which eventually led to death of macrophages [61].

### 3.3. ROS on Monocyte Recruitment

The circulating monocytes, which derive from hematopoietic stem cells in the bone marrow and migrate to peripheral blood, have the capacity of differentiating into tissue macrophages. This is generally considered as the major population of macrophages involved in pathophysiological development of human diseases. However, tissue macrophages have distinct mechanisms of hematopoiesis, and embryonic macrophages even bypassed monocytic stages [73]. The diversity in strain of macrophages was reflected by their name in particular organs and tissues, for example, Kupffer cells in the liver and microglia in the brain. It is a notion that, during disease progression that involves inflammatory response, inflammatory monocytes are developed and exhibited migratory capacity towards primary sites of the diseases. Deficiency of CX_3_CR_1_ in mice suppressed the activation of monocyte-derived macrophages in periphery and reduced macrophage infiltration and microglia proliferation, which subsequently attenuated brain ROS level and neuron apoptosis [74]. This gives rise to the notion that oxidative stress may have correlation with monocyte recruitment in human diseases. Indeed, it was found that H_2_O_2_ may serve as chemoattractant to monocytes, as evidenced by the observation in zebrafish larvae whose wound produced H_2_O_2_ to facilitate rapid macrophage recruitment [75]. Overexpression of UCP2, which relieved oxidative stress and intracellular ROS level of THP1 human monocytes, further reduced monocyte migration and adhesion through cellular monolayer [76]. Oxidative burst is therefore regarded as a favouring environment for monocyte activation. Previous study showed that ROS triggered CCL2-induced hyperalgesia in rats, which is attenuated in the presence of SOD confirming the role of ROS as facilitator in monocyte recruitment [77]. The authors also found that ROS level is not elicited in response to CCL2 in monocytes/macrophages with fewer expressions of CCL2 receptor, which indicated that intracellular ROS level may have an independent role in triggering recruitment of monocytes.

Moreover, metabolic stress may trigger expression of Nox4 in monocytes, increased intracellular H_2_O_2_ production, and thereby accelerated THP-1 monocyte migration [78]. The study also further explained why high fat diet-induced metabolic stress in mice had higher rate in monocyte chemotactic activity. The localization of Nox4 around focal adhesion and actin cytoskeleton of human-derived macrophages, together with the association with adhesion related proteins, supports the role of ROS in mediating macrophage motility [79]. The study also further postulated that ER stress stimulated THP-1 monocytes have augmented adhesion ability, and deletion of Nox4 blocked this activity. This further claimed that ER stress induced ROS production may be an endogenous factor in facilitating monocyte chemotaxis. Using Nox inhibitor apocynin or antioxidant catalase, recruitment of monocytes into atherosclerotic lesion was reduced, which suggested the central role of Nox-derived ROS in monocyte recruitment in atherosclerosis [80]. Very interestingly, ROS is associated with macrophage death (as discussed previously) driven by oxLDL and HDL in atherosclerosis; and also monocyte accumulation in diseased mice model proposes ROS as an important regulator for atherosclerosis progression.

Although the reported studies have highlighted ROS as a critical mediator of monocyte recruitment, the contribution of ROS as a chemoattractant of monocytes compared to others, as well as the factors that raise intracellular ROS in monocytes during pathophysiological process, remains unanswered. Previous study proposed that ROS-sensitized monocytes may have higher chemotactic response towards chemoattractants CCL5, CCL2, and PDGF-*β* [78], yet the study may not directly delineate the role of ROS in monocyte recruitment. There are few explanations for the difficulties of this investigation in deriving the cause-effect relationship: first, the reported models are insufficient to show the correlation of ROS and monocyte recruitment. Blocking ROS may drive the inhibition of other chemoattractants that further reduced monocyte motility; second, it is not clear whether increased monocyte recruitment is the consequences of enhanced monocyte proliferation and accumulation; thus the role of ROS in monocyte recruitment, accumulation, and proliferation needs to be established; third, monocyte is hypersensitive to ROS. Monocytes undergo extensive cell death in response to ROS in dose- and time-dependent manner [47]; proposed high levels of ROS are fatal to monocytes themselves. Therefore, the role of ROS in monocyte recruitment needs to be further deciphered.

### 3.4. The Role of ROS on Monocytes-to-Macrophages Differentiation

Studies have revealed that ROS drives monocytes-to-macrophages differentiation in* in vitro* culture of various types of monocytic cells, regardless of mouse or human origin. It was observed in human promyelocytic leukemia cell lines that production of ROS was induced in line with increased expression of macrophage marker CD14 during 1 alpha, 25-dihydroxyvitamin D (3) (VD3) induced differentiation. Regulation of monocyte differentiation is mediated through induction of ROS that further increases 5-lipoxygenase along with p38 MAPK activation [81]. In human promonocytic cell line U937, production of ROS was accelerated during macrophage differentiation induced by phorbol ester (PMA). This event was driven by NADPH oxidase, and it was observed that the persistent induction of Cox-2 along with monocyte differentiation was blocked by Nox inhibitors, suggesting the critical role of Nox in functional activation of proinflammatory gene Cox-2 [82]. Generation of ROS which serves as defense against invading microbes is regarded as the hallmark of monocyte/macrophage activation, though it is still not clear if ROS production directly results in macrophage maturation [47]. It was observed in the latter study that elimination of ROS by butylated hydroxyanisole (BHA) and other ROS inhibitors completely blocked monocyte/macrophage differentiation [83]. NADPH oxidase, in particular, is the molecule that primed to produce more ROS during macrophage differentiation. It is a typical ROS generator, and its expression as well as translocation to plasma membrane was induced by 1,25-dihydroxyvitamin D3 in human myeloid leukemia PLB-985 cell in line with increased surface markers CD11b and CD36 during macrophage differentiation [84]. The study also included* in vivo* intraperitoneal thioglycollate injection-induced mouse peritoneal macrophages model and findings showed that increased macrophage maturation is associated with enhanced cellular capacity in oxidizing LDL.

In lipopolysaccharide- (LPS-) induced macrophage differentiation model, it was observed that ROS production is essential in THP-1 differentiated macrophages for activation of HIF-1*α* and acquired adaptive ability in hypoxic microenvironment of inflammatory site [85]. It is therefore reasonable that high ROS level is associated with diseased environment, mainly because ROS facilitates the differentiated macrophages survival under hypoxic condition. A similar observation is made on 12-O-tetradecanoylphorbol-13-acetate- (TPA-) induced THP-1 cells in which activation of NADPH oxidase-derived ROS is associated with monocytic differentiation [86]. Interestingly, TPA was found to decrease expression of endogenous antioxidant enzyme catalase during human U937 macrophage differentiation. By applying catalase in TPA-treated monocytes, the differentiation process as well as ROS production was blocked [87], further confirming the role of ROS in mediating macrophage differentiation. By silencing the important endogenous redox homeostasis regulator NF-E2-related factor 2 (NRF2) in PMA-induced U937 cells, the ROS level is maintained at high level during macrophage differentiation and this was followed by higher expressions of proinflammatory cytokines [88]. Additionally, inactivation of oxidative stress-quenching molecule PPAR*γ* during saturated fatty acid-induced macrophage differentiation supports maintenance of high intracellular ROS level [89], and this process was found to involve an induced* de novo* synthesis of endogenous PPAR*γ* inhibitor ceramide [90]. Palmitate, the unsaturated fatty acid intervention, triggered ceramide production in macrophages and further derived the mitochondrial superoxide production that facilitates macrophage differentiation [91]. Interestingly, it was also reported that, in unprimed macrophages, differentiation towards alternative activated phenotype is ROS-dependent but not in classical activation of macrophages [83]. Consistent with the study, several studies also suggested that ROS-induced maturation of macrophages is associated with upregulation of proinflammatory gene expression [82, 88], the hallmark of classically activated macrophages. Though it is reasonable as macrophage is mainly present in inflammation site, this further renders the questions of correlation between ROS, inflammation, and macrophage maturation. Further discussion would be made in the latter section. Taken together, all these evidences have implied a reprogrammed redox homeostasis in differentiating macrophage from monocytes in terms of maintaining ROS level facilitating cell maturation.

## 4. Role of ROS in Macrophage Polarization

### 4.1. ROS Promotes M1

As mentioned above, M1 macrophages possess a high bactericidal function and defence against invading pathogens is the primary function of M1. To clear the site of injury, M1 macrophages tend to trigger the bactericidal response which involves the production of ROS and NO in contact with pathogen. The phagocytic function of M1 mainly depends on Nox2 gene, as discussed previously (Section 3.1.2). Production of ROS and NO by NADPH oxidase and nitric oxide synthase, in which both enzymes are generated from NADPH through pentose phosphate pathway [92], and Nox2 negatively regulates the phagosomal proteolysis [93]. It is also reported that M1 macrophages have reduced rate of acidification and proton-pumping activity compared to M2, which facilitates M1 macrophages to efficiently eliminate pathogens [94]. The effective microbicidal function of M1 macrophages requires continuous production of ROS followed by delayed maturation of phagosomes.* In vitro* stimulation of M1 macrophages with lipopolysaccharides (LPS) promotes recognition by TLRs, the primary LPS receptor, and, occasionally, LPS binds to phagocytic MAC1 receptor independent of TLRs [95]. The association of LPS with receptors drives the production of ROS and genes alterations. LPS-induced ROS generation is Nox-dependent and it further supports the ROS-induced TNF*α* production, which is evidenced from the reduced TNF*α* in PHOX^−/−^ mice [96]. Although performed in human embryonic kidney cells HEK293T, previous study also proposed that LPS-induced ROS production is regulated by the direct association between cytoplasmic tail of TLR4 and COOH-terminal of Nox4. RNA interference against Nox4 on TLR4 expressing cells blocked LPS-induced ROS production [97]. Besides, ROS may serve as secondary messenger in the LPS-induced signal transduction, facilitating the regulation of downstream pathways such as mitogen-activated protein kinase (MAPK) and NF-*κ*B [98]. Activation of these pathways by H_2_O_2_ promotes expression of proinflammatory genes.

Upon being challenged with TLRs ligands, activation of TLRs binding on macrophages triggers the translocation of TRAF6 from TLR signalling complex to evolutionarily conserved signalling intermediate in Toll pathways (ECSIT) on outer mitochondrial membrane that primes the generation of mitochondrial ROS and phagocytosis activity [99]. The stimulation of mitochondrial ROS production via electron transport chain in the inner mitochondrial membrane facilitates macrophage reprogramming towards M1 phenotype. It is evidenced by the murine model with overexpressing catalase in mitochondria, which showed increased bacterial loads after infection compared to wild type. Another study by Infantino et al. also links ROS production to homeostatic function of mitochondria in macrophages, in which it suggested that the mitochondrial citrate carrier that functions in transporting citrate into cytoplasm exerts important function in mediating ROS generation upon LPS induction. By either transient deactivation of citrate carrier or using citrate carrier inhibitor, BTA attenuated the production of nitric oxide, ROS, and prostaglandin. And the notion may be contributed by the acetyl-CoA and oxaloacetate, the cleavage product of citrate required for the production of ROS [100]. The production of mitochondrial ROS was also further explained to be mediated by immunoresponsive gene 1 (IRG1), which improved oxidative phosphorylation and thereby increased ROS production in phagosomes. Using zebrafish infection model, the study demonstrated that depletion of IRG-1 in macrophage lineage cells failed to employ fatty acid as their fuels and leads to impaired ROS production and bactericidal activity [50]. Using genetic deletion of p47^PHOX^ and gp91^PHOX^, as well as apocynin, the NADPH inhibitor promoted the phenotypic changes of microglial towards M2-like phenotype and increased the M2-like genes response, which further evidenced the role of NADPH oxidase in maintaining the phenotype of M1 [101].

M1 macrophages activation is always correlated with upregulation of TNF*α* mediated inflammatory response. Activation of TNF*α* is deemed to depend on interaction of TNF with TNF receptors that triggers the downstream signalling, mitogen-activated protein kinases (MAPK) and I*κ*B-kinases (IKK), that activates NF-*κ*B signalling [98]. It is reported that H_2_O_2_ tends to accumulate in NF-*κ*B deficient cells when exposed to TNF; the H_2_O_2_ further oxidized the catalytic cysteine of MAPK phosphatases and triggered activation of MAPK cascades including JNK and p38 MAPK [102]. Excessive H_2_O_2_ also promotes I*κ*B-kinase activation and drives tyrosine phosphorylation of I*κ*B*α*, leading to stimulation of NF-*κ*B signalling [103]. Recent studies postulated that the macrophage reprogramming towards M1 phenotype along with proinflammatory gene expressions by small molecules is mediated by activation of MAPK and NF-*κ*B signalling cascades [104, 105], though involvement of ROS was not mentioned. Given that ROS is closely related to the activation of MAPK and NF-*κ*B, ROS may partially regulate macrophage polarization towards M1. SIRT2, a NAD-dependent histone deacetylase, was also found to be involved in LPS-induced ROS generation in macrophages; deletion of SIRT2 inhibited NF-*κ*B p65 nuclear translocation and M1 related gene expressions [106]. Moreover, Nox-derived hydrogen peroxide H_2_O_2_ was believed to be the major ROS in response to microglial activation [107], as evidenced from the observation that catalase blocked the MAPK and NF-*κ*B mediated LPS-induced proinflammatory genes expressions, but not superoxide dismutase [108].

Rowlands et al. have postulated that TNF*α* in circulation increased mitochondrial Ca^2+^ and thereby triggers the endocytosis of TNF*α* receptor 1 mediated by TNF*α* converting enzyme TACE. The inflammatory response is further regulated through stimulation of mitochondrial complexes to generate ROS and binding of unattached TNF*α*R1 to soluble TNF*α*. It is a negative feedback loop in responding to TNF*α* induced inflammation, in which overexpression of catalase blocked mitochondrial H_2_O_2_ dependent TNF*α*R1 shedding and thus enhanced the inflammatory response [109]. This study is performed on lung endothelium, while another recent study also demonstrated the similar regulation on a mucin glycoprotein MUC1 expressed on alveolar macrophages. MUC1-expressing M1 macrophages activation increased MUC1 ectodomain shedding in TACE dependent manner; the upregulation of MUC1 is associated with blunted ROS production and phagocytic activity in M1 macrophages [110]. The findings also further revealed that MUC1-deficient M0 macrophage has augmented ROS and TNF*α* secretion, suggesting the tight regulation of ROS homeostasis in macrophages for maintaining the proper phagocytic activity and inflammatory response. The uncoupling protein 2 in inner mitochondrial membrane also interferes with ROS production; downregulation of UCP2 by LPS in murine bone marrow derived macrophages promotes proinflammatory cytokine secretions [111].

**Figure 1:**
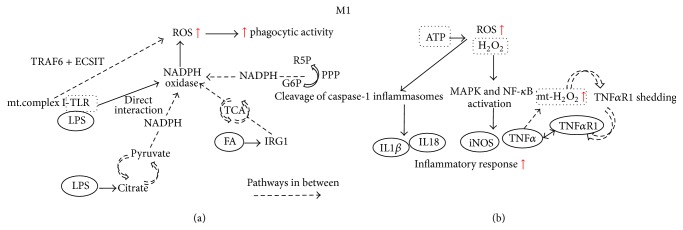
Involvement of ROS in regulating M1 responsible phagocytic activity and inflammatory response. (a) Multiple pathways are involved in generating NADPH, followed by ROS production by NADPH oxidase. The high ROS level mainly used to mediate the phagocytic activity of M1 macrophages. (b) ROS serves as second messenger mediating the inflammatory response of M1 macrophages, primarily through MAPK and NF-*κ*B as well as inflammasome activation. Mt, mitochondrial; FA, fatty acid; G6P, glucose-6-phosphate; R5P, ribulose-5-phosphate; PPP, pentose phosphate pathway; TCA, tricarboxylic acid cycle.

Besides, the activation of inflammasome followed by ROS production has been implicated in regulating proinflammatory cytokines, IL-1*β* and IL18 production; it requires TLR ligands such as LPS for gene synthesis and second stimulus produced by DAMP for cleavage of caspase-1, which further stimulates the protein secretions [112]. The activation by second stimulus such as ATP will trigger ROS generation, followed by caspase-1 and inflammasome activation and cytokine production. Early study postulated that NADPH-derived ROS is responsible for the upstream of inflammasome activation. It is evidenced from the blockade of caspase-1 and cytokine secretion after addition of DPI, flavoprotein inhibitor of NADPH oxidase, which suggests that the interaction of ROS with inflammasome exerts important function for proinflammatory cytokines production [113]. However, there is contradicting study suggesting that superoxide dismutase 1, the antioxidant, also regulates caspase-1 activation [114]. Apart from that, mitochondrial derived ROS may also activate inflammasomes, in which the notion is further substantiated by addition of rotenone and antimycin A; the respiratory chain inhibitors increased ROS production followed by IL-1*β* secretion [115]. Deletion of dynamin-related protein 1 and the mitochondrial fission protein indirectly influences the localization of NLRP3 inflammasome, leading to caspase-1 activation and IL-1*β* production [116]. Though ROS may be crucial for inflammasome activation and priming, there is also explanation proposing that redox signalling in macrophage may be derived from other cell types, and the inflammasome activation depends on the redox status in particular to cell types upon PAMP stimulation. In healthy macrophages, the antioxidant system is stimulated to counteract the high level of ROS and impaired antioxidant response leads to low inflammasome activation and IL-1*β* production [117]. Nonetheless, redox signalling in inflammasome activation is far more complicated as postulated and studies reported on caspase-8-dependent inflammasome activation and ROS-induced NLRP3 inflammasome priming have been further proposed recently [118, 119]. Yet, there is no definite answer on how ROS impacts inflammasome activation or priming and more studies are needed to further justify the mechanisms involved. The mechanisms involved by M1 macrophages are illustrated in Figure 1.

### 4.2. ROS Promotes M2

Depending on the content of intracellular glutathione, the M1 and M2 macrophages are characterized as oxidative and reductive macrophages, suggesting the redox regulation in macrophages physiology [120]. In contrast to M1 macrophages, M2 activation stimulates increased arginase-1 activity and is accompanied by reduced ROS and NO generation. The functions of tissue remodeling and wound healing of M2 macrophages are explained to be attributed by the macrophages effect in expressing increased cathepsin S and cathepsin L and reduced NAPDH oxidase (Nox2) activity, which all improved the phagosomal proteolytic activity of M2 (IL-4) macrophages. Reduced Nox2 also improved the wound healing functions of M2 in degrading disulphide protein [121]. Furthermore, the interaction between M2 macrophages with apoptotic bodies triggers instability of NADPH oxidase Nox2 mRNAs through binding blockade of RNA-binding protein SYNCRIP to Nox2 3′-UTR. And this further defects the ROS production and leads to M2 macrophage polarization [122]. In type I diabetes NOD murine model, the deficiency of NADPH oxidase-derived superoxide has rendered the skewing of islet resident macrophages towards M2 phenotype followed by downregulation of TNF*α* and IL1*β* in surrounding environment and further protects *β*-cell from destruction [123]. Besides, the mutation of cytosolic protein of Nox2, p47^phox −/−^, also favours the macrophage reprogramming towards M2 phenotype together with upregulation of arginase-1, Ym1, and Fizz1 [124]. The notion is also further evidenced in diseased state of microglia, where deletion of p47^phox^ potentiates macrophages towards M2 upon LPS stimulation followed by increase of M2 markers IL-4R*α*, Ym1, Fizz1, Mrc1, CD163, and MARCO. Addition of apocynin, the inhibitor of NADPH oxidase, also gives similar trend of outcome and the effect is reversed upon intervention of IL-4 neutralising antibody [101]. All these further propose depletion of Nox2 accompanied by ROS reduction which is important for reprogramming of M1 to M2 phenotype.

Apart from the suggested role of NADPH oxidase in regulating M2 macrophages, superoxide dismutase (SOD) enzyme, which catalyses superoxide dismutation and was associated with H_2_O_2_ production, also contributed to M2 macrophages activation. Study by He et al. postulated that Cu, Zn-SOD^−/−^ mice had abundance of alveolar macrophages in M1 phenotype, while Cu, Zn-SOD^TG^ mice predominantly expressed M2 macrophage markers. The function of Cu, Zn-SOD in modulating M2 alternative activation is regulated by redox-dependent STAT6 translocation [125]. Indeed, previous study revealed the absence of IL-4 related genes expressions in STAT6 deficient mice upon stimulation by IL-4. Silencing of STAT6 in T lymphocytes showed incapability to polarize to Th2 phenotype in the presence of IL-13 or IL-4. This also further postulated that STAT6 is implicated for M2 macrophage polarization, given that M2 macrophages have the same differentiation manner as Th2 lymphocytes [126]. Moreover, as opposed to M1 macrophages, extracellular ATP blocks IL-1*β* in M2 macrophages. This was claimed to be mediated by two mechanisms, the direct blockade of ROS and inflammasome trapping through clustering of actin filaments, which are both associated with reduced ATP plasma membrane ion channel, P2X_7_R [127]. Recent study also postulated the glucose metabolism related protein carbohydrate kinase-like protein (CARKL) promotes M2 activation. Reduced pentose phosphate pathway (PPP) flux in M2 macrophages is mediated by CARKL that catalyses the formation of sedoheptulose-7-phosphate, the intermediate of PPP. The reduced glucose metabolism activated M2 was evidenced by addition of metformin and rotenone which blocked (IL-4) M2 genes expressions. The study also further observed reduction in NAD by not NAD^+^ levels which suggested that CARKL may be important in regulating the redox balance in glucose metabolism [17].

**Figure 2:**
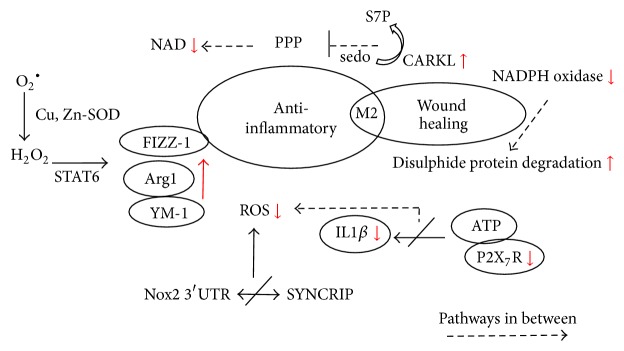
Involvement of ROS in regulating M2 responsible inflammation resolving and wound healing activities. Multiple pathways are involved in reducing NADPH and its oxidase followed by reduced ROS generation. The low ROS level was accompanied by reduced inflammatory mediators; increased M2-regulated genes responsible for inflammation resolution; and increased disulphide protein degradation which enhanced wound healing effect of M2. S7P, sedoheptulose-7-phosphate; sedo, sedoheptulose; PPP, pentose phosphate pathway; SYNCRIP, synaptotagmin binding, cytoplasmic RNA interacting protein; SOD, superoxide dismutase.

Although impact of redox signalling or ROS production in M1 macrophages activation seems to interfere with M2 macrophage priming, study by Zhang et al. postulated that ROS production is also important for M2 macrophage differentiation. Intervention of antioxidant butylated hydroxyanisole BHA by inhibiting Nox-mediated O^2−^ production before differentiation by M-CSF treatment blocked monocyte differentiation to M2, which suggests that ROS may implicate the early stage of M2 macrophage differentiation. Further intervention of BHA in urethane-induced murine lung cancer model also attenuated the occurrence of tumor-associated macrophages and thereby reduced tumor progression [83]. Also, study revealed that KLF4 triggers MCP-1-induced protein (MCPIP) to stimulate ROS production in IL-4-induced M2 macrophages and ROS attenuation blocked ER stress in M2 macrophages. Removal of the MCPIP shifted the macrophage phenotype towards M1 with increasing phagocytic function [128]. These studies may render questions for activation and priming of alternative M2 macrophages, whether ROS may impact different stages of M2 macrophage manifestation, which need to be further investigated. The mechanisms involved by M2 macrophages are illustrated in Figure 2.

## 5. Discussion

### 5.1. ROS-Controlled Macrophage Polarization in Disease Progression: A Potential Drug Target?

More evidences postulated that macrophage polarization played critical role in initiation and progression of multiple human diseases, as described above. In some cases, ROS plays a critical part in triggering disease-specific skewing of macrophages. This has been particularly observed in tumorigenesis and atherosclerosis in which the dysregulation caused by oxidative stress and inflammation have been extensively studied. However, the involvement of oxidative stress in diseases progression may be very broad and results in pathogenesis linking variety levels of biological processes; direct cues on ROS-directed macrophage polarization further dominate diseases progression which are still lacking. This is also not justified due to the nature of macrophages as effectors cells, whose function in part is antigen presenting. In this case, ROS-driven macrophage polarization is thus far difficult to become putative drug target owing to the lack of specificity. This shortage is reflected in multiple levels, and pharmaceutical companies have to develop systems that particularly target macrophage ROS as well as its polarization. This includes not only drug target study but also development of drug delivery system. Interestingly, recent studies have revealed that liposome specifically delivered molecules to macrophages [129], which allows molecules to be specifically targeted on macrophages in the body. And development of high-resolution transcriptome analysis allows differentiation of M1 macrophages from M2 phenotype by specific surface markers [130]. Development of particular antibodies also allows recognition of individual subtype of macrophages in human body. Potential molecules targeting ROS signalling and macrophage reprogramming could be further enriched in order to discover the target treatment. More experimental investigations as well as clinical trials shall be conducted to prove the hypothesis.

### 5.2. Antioxidative Herbal Supplements Regulating Macrophage Polarization: Any Clues for Diseases Treatment?

There are a plenty of herbal supplements available for the indication of health improvement. Herbal supplements are commonly employed, by people not only in Asian countries with tradition of using herbal remedy for diseases treatment, but also in countries where use of herbal products is under strict control and closed monitoring, for example, Europe and US. There are an increasing number of populations who favour herbal products as dietary supplements due to the concrete health-improving effect of herbal supplements as demonstrated from the scientific evidences by both benchtop and clinical studies. Most of these herbal supplements, such as* Ginkgo biloba*, lingzhi mushroom, baicalin, and some composite herbal formulae, exhibit excellent antioxidative effect in laboratory studies. Indeed, herbal supplements generally contain a series of flavone and phenol-like compounds that work as effective scavengers of ROS and that confer the health-improving effects. Interestingly, recent evidences demonstrated that some herbal supplements could regulate macrophage polarization in preclinical models of human diseases. Study by Lam et al. proved that PHY906, an herbal adjuvant derived from ancient Chinese Medicine formula Huangqin Decoction, has beneficial effect to cancer treatment. The tumor regression effect of PHY906 may be associated with its regulation on polarization of macrophage within tumor microenvironment. Tumor from mice receiving PHY906 showed more infiltrated M1 macrophages which facilitates tumor cells killing activity [131]. Our previous study on baicalin, which is the major compound in PHY906 and is used as calming and soothing supplement, could reskew M2 polarized macrophages towards M1 phenotype. This effect was reflected in tumor microenvironment, with more M1 but reduced M2 macrophages observed after baicalin treatment. Removal of macrophages attenuated tumor inhibition by baicalin [132]. Interestingly, baicalin is generally used to reduce inflammation. The property of baicalin and baicalin-containing herbal supplement in activating proinflammatory phenotype of tumor-associated macrophages may underscore a variety of mechanisms underlying effect of baicalin on macrophages. In fact, baicalin has been reported as a ROS generator as well as scavenger in macrophages [133, 134]. Given the complicated role of ROS as we discussed above, the dual role of baicalin in ROS homeostasis may be involved in baicalin-mediated proinflammatory macrophage skewing. More scientific evidences have to be acquired in order to prove if ROS involves baicalin-mediated macrophage functions, but this also further raises the consideration on the scientific scrutiny in consumption of herbal products as daily dietary supplements, assuming that they may impact ROS-driven macrophages polarization. Similar cases are reported in other antioxidative herbal supplements, including bitter mushroom [135] and lingzhi mushroom [136], which facilitates the regulation of macrophage polarization towards particular phenotype. Nonetheless, the function of polarized macrophages with particular phenotype varies across different diseases, and there is no justifiable conclusion on the function of each individual phenotype of macrophages in all diseases. In this case, herbal supplements consumption without concerning their use in particular diseases may cause severe consequences, at least in the context of macrophage polarization. As we still could not deny the drug-like effect of many herbal supplements, their proper use shall be further supported by more scientific evidences.

## 6. Conclusion

In summary, macrophages are majorly diversified into two distinct phenotypes. A variety of peripheral and residence factors may determine the dominant phenotypes of macrophages within tissues. Although it is not yet concluded whether this polarization of macrophages is* in situ* reversible, it has been shown that the overall shift of macrophage phenotypes plays a critical role in the progression of various human diseases. Instead of merely being a harmful byproduct of metabolism, ROS has been shown to get involved in the functional and phenotypic regulation of macrophages. ROS is able to control the cell death, proliferation, motility, and phagocytic ability of macrophages. Intriguingly, it is recently observed that ROS may play a complicated role in regulating macrophage polarization, which implies the potent future therapeutic approaches for life-threatening diseases. Development of target-specific delivery system has been supportive for drug development, and natural antioxidants with immunomodulatory function shall be considered. More scientific evidences as well as clinical trials are imperative and emerging.
